# Effects of Ticket-Checking Failure on Dynamics of Pedestrians at Multi-Exit Inspection Points with Various Layouts

**DOI:** 10.3390/ijerph16050846

**Published:** 2019-03-08

**Authors:** Meiying Jiang, Qibing Jin, Lisheng Cheng

**Affiliations:** 1 School of Information Science and Technology, Beijing University of Chemical Technology, Beijing 100029, China; jmy@mail.buct.edu.cn (M.J.); jinqb@mail.buct.edu.cn (Q.J.); 2 School of Humanities and Law, Beijing University of Chemical Technology, Beijing 100029, China; 3 School of Mechanical and Electrical Engineering, Beijing University of Chemical Technology, Beijing 100029, China

**Keywords:** ticket, check-out, competitive behavior, subway station, multi-exit, cellular automata, simulation

## Abstract

It is of great significance to understand the dynamics and risk level of pedestrians at the multi-exit inspection points, since they are the bottlenecks of pedestrian flow leaving public places, such as subway and railway stations. Microscopic simulations were carried out with a cellular automata model to investigate the effects of ticket-checking failure on pedestrian dynamics when passing through the multi-exit inspection points with parallel, convex and concave layouts. It was found that although ticket-checking failure could reduce the passing efficiency, it also lowers the competitive level between pedestrians and enhances passing safety in the range of medium and high pedestrian density. The competitive level decreases when increasing the probability of ticket-checking failure and the corresponding delay. The probability of ticket-checking failure and the corresponding delay have equivalent effects on passing efficiency and safety, and can be integrated as average delay. A fitted equation was proposed for the dependence of passing efficiency and safety on average delay. With the existence of ticket-checking failure in reality, the concave layout of the multi-exit inspection points gives rise to a much lower competitive level compared with the parallel and convex ones, which would enhance the safety of pedestrians at the exits.

## 1. Introduction

In traffic hubs, such as metro and subway stations, public safety and health is a major concern in their operation management [1,2]. The specific issues include air quality [3,4], crowd management [5,6,7], and emergency evacuation in sudden events [8], such as fire disasters [9,10] and terrorist attacks [11,12]. Among these issues, both crowd management and emergency evacuation are closely associated with the pedestrian dynamics of crowds in the station, especially in big cities of China, where there is a large population and subways play a significant role in public transportation.

In the station, the inspection points used for checking out are usually placed at the exits. Such positions are generally the bottleneck of pedestrian flow, where there is a relatively high density of pedestrians and even congestion of pedestrians [13,14]. In a crowd with a high density, there are more intensive interactions and higher probabilities of collision between pedestrians than in a low-density situation [15]. Moreover, there would be competition for paths of moving ahead between the pedestrians [16]. These factors would definitely increase the risk of conflicts, disturbance, crushes and stampede. Joseph et al. [17] reported that a human stampede is the largest one among the potential health risks in a pedestrian crowd situation. In fact, the risk of stampede accidents itself is very high in the abnormally crowded subway stations of China [6]. In addition, for the sake of increasing flowrate, several ticket-checking machines are usually used to form multiple exits. The use of multiple exits would further increase the complexity of pedestrian flow, since it will introduce the behavior of exit selection and reselection [18]. The exit-selection behavior would give rise to the redistribution of pedestrians and the change of local crowd density [18,19,20,21]. The layout of the multiple exits also influences the dynamics of pedestrians. Yue el al. [22] and Zhao et al. [23] found that symmetric multiple exit layouts have higher evacuation efficiencies, compared to asymmetric ones. In our previous work [24], we found that the layout of the multiple exits has significant effects on the dynamics of pedestrians in an evacuation process, and the concave layout allows a higher passing efficiency in the high crowd density range than the parallel and convex ones. Therefore, the dynamics of crowds at multi-exit pedestrian flow bottlenecks has drawn great public health concerns and research interest for the sake of optimizing pedestrian management [24,25].

In real situations, there is a certain probability of ticket-checking failure during the process of checking out with multi-exit inspection machines. Such events may be caused by mechanical faults, no preparation of an integrated circuit (IC) card in advance, failure of the IC card, response delay of checking-out mobile applications, pedestrians being unfamiliar with the procedure for checking out with a ticket, delay of near field communication (NFC) between the mobile phone and the inspection sensor, and so on. A ticket-checking failure will not only cause local pedestrians to wait and delay passing through the exits, but also interrupt the pedestrian flow. Both these aspects greatly affect the movement and exit selection of the follow-up pedestrians, and may even lead to the formation of pedestrian shockwaves [26]. Therefore, the behavior of ticket-checking failure has complicated effects on the process of passing through multi-exit inspection points.

Efforts have been made to understand the ticket-checking process, since it is helpful for the enhancement of passing safety and the development of emergency plans. Tang et al. [27,28] investigated the pedestrian movement at the hall of a high-speed railway station during the check-in process with a cellular automata (CA) model. They found that the passenger’s arrival rate to the hall and the service efficiency of ticket barrier have significant effects on the complex phenomena occurring in the hall, the boarding efficiency and the number of passengers in the hall during the check-in process. Wang et al. [29] studied the pedestrian flow in a station hall during the Chinese Spring Festival travel rush by carrying out microscopic simulations with a social force (SF) model. They considered the effects of passenger number, ticket checking patterns (automatic or manual), baggage volume, and anxiety on passing efficiency. They found that automatic ticket-checking allows the crowd to move forward in an ordered manner, promising lower risk of casualty than the manual one. Zheng et al. explored the queuing behavior of pedestrians during security checks in the subway station.

However, there are few reported works that investigate the effects of ticket-checking failure in the process of checking out and passing through the exits, so the effects of ticket-checking failure on the dynamics of pedestrians at multiple exits, which are critical to enhancing the passing efficiency and safety, are still unclear. Understanding the complex behaviors of pedestrians when passing through multi-exit inspection points from a macroscopic view is a challenge. Therefore, in this work, we carried out microscopic simulations of pedestrians passing through multi-exit inspection points with various layouts to understand the effects of ticket-checking failure on the dynamics of pedestrians.

## 2. Model and Simulation Details

### 2.1. Cellular Automata Model

**Figure 1:**
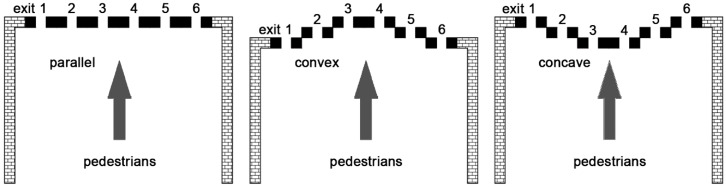
Schematic of the three layouts of multi-exit inspection points: parallel, convex and concave. The inspection machines are shown in black.

Several models are available for the simulation of crowd evacuation and pedestrian dynamics, such as the fluid dynamics model [30], SF model [31,32] and agent-based model [33]. Following our previous work [24], here we adopt the CA model for the microscopic simulations, since it not only has high computational efficiency, but it is also able to consider various factors by incorporating their effects into particular rules. In the CA model, the scene was treated as two-dimensional grids, where each cell has two states, being empty or occupied by a pedestrian or a building fence. In a timestep, a pedestrian can move to an adjacent cell or not move. Here the behavior of ticket-checking failure and pedestrian dynamics associated with only three symmetric layouts of inspection points, namely parallel, convex and concave, were considered, since an asymmetric layout would give rise to non-balanced effects during the evacuation of pedestrians (see Figure 1) [22,23,34].

In general, the CA model uses a static floor field to represent the tendency of pedestrians moving towards exits. The behavior of multi-exit selection is affected by various effects, such as building structures, pedestrian psychology, evacuation signs, and so on. However, following our previous work [24], here we consider only two main aspects that determine the exit selection behavior, the spatial distance of pedestrian i from the exit m, and the local density of pedestrian in front of the exit. Similar to previous work of Yuan et al. [35] and Zheng et al. [36] about the calculation of escape probability, the spatial distance from pedestrian i to exit m is calculated as follows [24]:

(1) $$r_{i}^{m}=\sqrt{{\left(x_{i}-x_{m}\right)}^{2}+{\left(y_{i}-y_{m}\right)}^{2}}$$

If there are no pedestrians near the front of the exit, the probability of pedestrian i selecting exit m as the target would increase when $r_{i}^{m}$ decreases. The local density of pedestrian near the front of exit m is calculated as:

(2) $$d_{i}^{m}=\frac{\sum_{j=1}^{w}c_{j}^{m}}{w}$$

where w is the number of cells near the front of exit m, and $c_{j}^{m}$ represents the state of a cell with 1 or 0 representing being occupied or not. If the spatial distance of the pedestrian to the exit is not considered, the probability of selecting exit m increases when $d_{i}^{m}$ decreases.

When taking into account only the distance or density effect, the probabilities of exit selection can be respectively calculated as:

(3) $$p_{i-r}^{m}=1-\frac{(k-1){\left(r_{i}^{m}\right)}^{k_{r}}}{\sum_{i=1}^{k}{\left(r_{i}^{m}\right)}^{k_{r}}}$$

(4) $$p_{i-d}^{m}=1-\frac{(k-1){\left(d_{i}^{m}\right)}^{k_{d}}}{\sum_{i=1}^{k}{\left(d_{i}^{m}\right)}^{k_{d}}}$$

where $k_{r}$ and $k_{d}$ are constants, whose proper values of 0.5 and 1.2 had been verified in our previous work [24]. Taking into account both the distance and local pedestrian density, the probability of pedestrian i selecting exit m can be calculated as:

(5) $$p_{i}^{m}=\frac{\alpha p_{i-r}^{m}+\beta p_{i-d}^{m}}{\alpha +\beta }$$

where α and β are the weights of distance and local pedestrian density, respectively. They can be calculated as:

(6) $$\alpha =\frac{1}{k}{\sum_{i=1}^{k}\left|1-\frac{k_{r}{}_{i}^{m}}{\sum_{i=1}^{k}r_{i}^{m}}\right|}^{k_{\alpha }}$$

(7) $$\beta =\frac{1}{k}{\sum_{i=1}^{k}\left|1-\frac{k_{d}{}_{i}^{m}}{\sum_{i=1}^{k}d_{i}^{m}}\right|}^{k_{\beta }}$$

where $k_{\alpha }$ and $k_{\beta }$ are the scalar constants associated with the importance of distance and local pedestrian density, respectively. Since the effects of distance and local pedestrian density have been converted into probabilities, both $k_{\alpha }$ and $k_{\beta }$ were set to 0.5.

Following our previous work [24], a sub-static floor field was used to consider the interaction of pedestrians near the front of exit. Pedestrian i selects exit m as the target to move toward according to $\max\{p_{i}^{m}\}$, and then determines the cell for the next step according to the sub-static floor field. The rules for the assigning of the sub-static floor field are as follows: the cells of exit are assigned 0, those of building fence and inspection machines are assigned ∞, and the left cells are assigned by the distance from the exit. 

The event of ticket-checking failures is determined by a random number between 0 and 1. When the random number is smaller than the given failure probability, the pedestrian fails to check a ticket. The delay by checking failure is determined by a normal distribution with a given value as average and a standard deviation of 10% from the average.

The simulation runs according to the following procedures: Step 1: Calculate the static floor field for the inspector area. Step 2: Calculate the selection probabilities from the pedestrian to each exit, and then confirm an exit as the target. Step 3: Calculate the sub-static floor field with the target exit, and then confirm a target cell. Step 4: Determine whether the pedestrian on the front of exit fails to check the ticket. If yes, the corresponding pedestrian will stay unmoved for a given period of time, and then recheck and pass through the exit. Step 5: Update the locations of all pedestrians basing on their target cells. Step 6: End the simulation when time runs out, otherwise return to Step 2.

### 2.2. Simulation Details

The scene of simulation contains six inspection points with six exits, and is composed of 30 × 30 cells. Each cell has a size of 0.4 × 0.4 m^2^, which is approximately the area occupied by a pedestrian. In the initial configuration, pedestrians are uniformly and randomly distributed in the scene. The moving speed of the pedestrian is about 1 m/s, so the timestep was set to 0.4 s. The average density of pedestrian in the scene is calculated as:

(8) $$\rho =\frac{N}{A}$$

where *N* is the number of pedestrians and *A* is the area of the scene. In the simulation process, if there are pedestrians leaving from the exit, these pedestrians would be added into the simulation system randomly at the end of the scene to maintain the average density. The total timestep of the simulation is 20,000, where 1000 steps are used for equilibration and 19,000 steps are used for sampling. The initial configuration may have an obvious effect on the dynamics of pedestrian [14], however, a period of 400 s is sufficient to avoid such effects. The average density of pedestrians ranges from 0.025 to 5.5 persons/m^2^, the failure probability ranges from 0 to 0.25, and the considered delay is 0 to 10 s, considering the situation in the subway stations of Beijing.

## 3. Results and Discussion

### 3.1. Passing Efficiency

#### 3.1.1. Effects of Pedestrian Density on Flowrate

**Figure 2:**
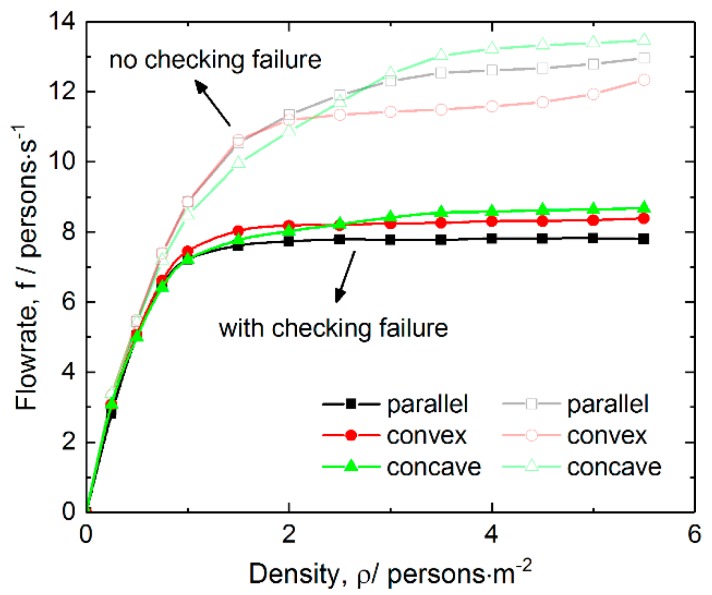
Total flowrate as a function of average pedestrian density in cases with and without checking failure for the three layouts of inspection points. The probability and average delay are 0.08 and 3 s, respectively.

We first compared the dependence of pedestrian flowrate on average pedestrian density in cases with and without ticket-checking failure. Figure 2 shows the total flowrate as a function of average pedestrian density in cases with checking failure for the three layouts of inspection points. For comparison, the reported results of those without checking failure are also shown in figure [24]. The results reveal that the flowrate increases rapidly in the density range of 0–1 persons/s, but then becomes saturated at a density of about 2 persons/s. This is because the flowrate mainly depends on the number of pedestrians arriving at the exit per unit time, and the number is proportional to the density. However, when increasing the density, the flowrate is increasingly dominated by the speed of pedestrians passing through the exit. This is similar to the two-stage queuing process reported in previous work that the pedestrian first selects and walks toward a queue, and then enters and waits in that queue [37]. With the existence of ticket-checking failure, the corresponding pedestrians delay passing through the exit because of wondering and rechecking, thus the flowrate decreases. Even though the pedestrians reach a higher density, the flowrate cannot be increased. Therefore, although the failure probability and delay are as small as 0.08 and 3 s, respectively, the saturated flowrate is reduced by 30%, and the corresponding density is also decreased to 1.5 persons/m^2^ from about 4 persons/m^2^. In addition, in the medium and high-density range, the concave layout of inspection points has the largest flowrate among the three layouts, which is similar to the case without checking failure. The flowrate for the concave layout is 1 persons/s larger than that of the parallel layout.

#### 3.1.2. Effects of Failure Probability on Flowrate

**Figure 3:**
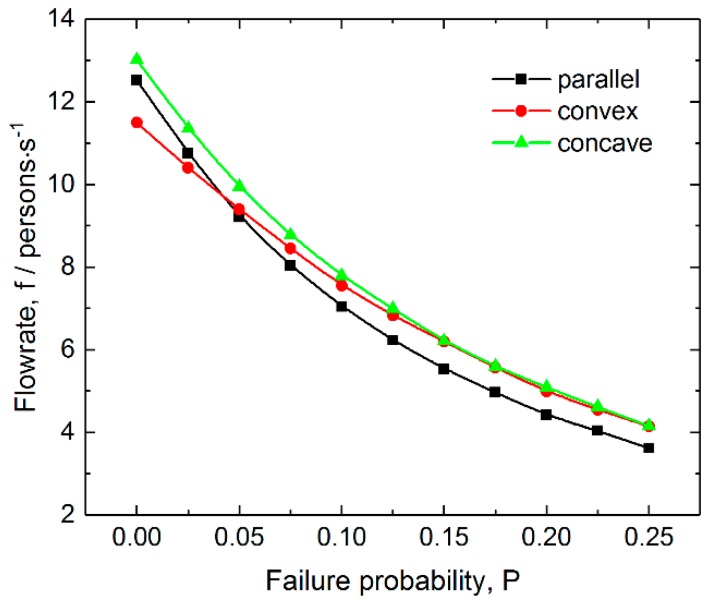
Total flowrate as a function of checking failure probability for the three layouts of inspection points. The density of pedestrian and delay are 3.5 persons/m^2^ and 3 s, respectively.

The behavior of ticket-checking failure was considered from two aspects: failure probability and delay. Figure 3 shows the flowrate as a function of failure probability for the three layouts. It can be seen from the figure that when the average pedestrian density and delay are 3 persons/m^2^ and 3 s respectively, the flowrate decays nonlinearly when the failure probability increases. This is because an increasing failure probability would increase the frequency of delays at the front of the exit. The figure also indicates that the flowrate for the parallel layout decreases most rapidly when the failure probability increases, which suggests that this layout has the weakest resistance to ticket-checking failure in terms of passing efficiency. In addition, the concave layout has the maximal flowrate in the whole range of failure probability.

**Figure 4:**
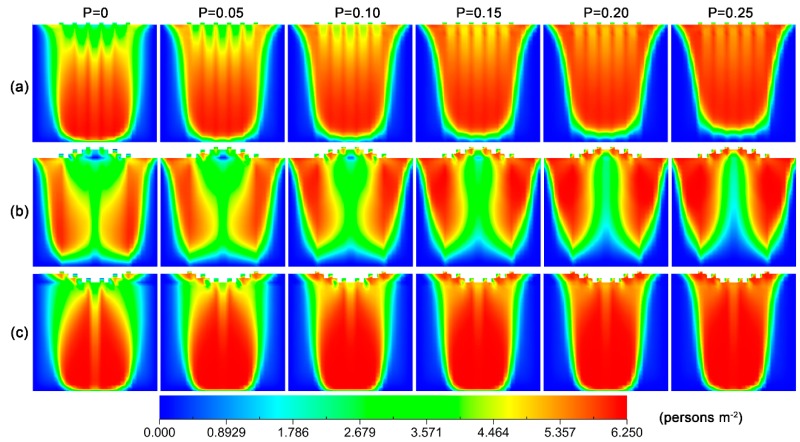
Density distribution of pedestrians in the scene at different checking failure probabilities for the three layouts of inspection points: (**a**) parallel, (**b**) convex and (**c**) concave. The density of pedestrian and delay are 3.5 persons/m^2^ and 3 s, respectively. The unit of pedestrian density is persons/m^2^.

To get an insight into the effect of failure probability on flowrate, we calculated the density distribution of pedestrians for various failure probabilities, which is shown in Figure 4. 

The figure reveals that when there is no checking failure, pedestrians are able to pass through the exits quickly, and a low-density region forms near the exits. However, with the increase in failure probability, the checking-failure pedestrian would be held up at the exit, resulting in the congestion of pedestrians. For the convex layout, there is a low pedestrian density region near the middle front of exits in the low probability range, and the area of this region decreases when the probability of checking failure increases. Such a distribution occurs because the passing process is composed of two steps: the first step is to move to the front of exits and the second step is to pass through the exit. Pedestrians tend to select the side exits as targets, since they are closer. This tendency would give rise to the gathering of pedestrians at the lateral regions and prevent pedestrians from approaching the middle exits. When the failure probability is greater than 0.15, such a region nearly disappears. This phenomenon is because the second step is interrupted by an increasing probability, and the pedestrians are not able to pass through the middle exits as soon. For the concave layout, in the low probability range, the distribution of pedestrian density has low-density regions in the two lateral parts near the front of the exits. The area of these two regions also decreases when an increasing probability of checking failure. When the probability is above 0.1, these regions disappear.

#### 3.1.3. Effects of Delay on Flowrate

**Figure 5:**
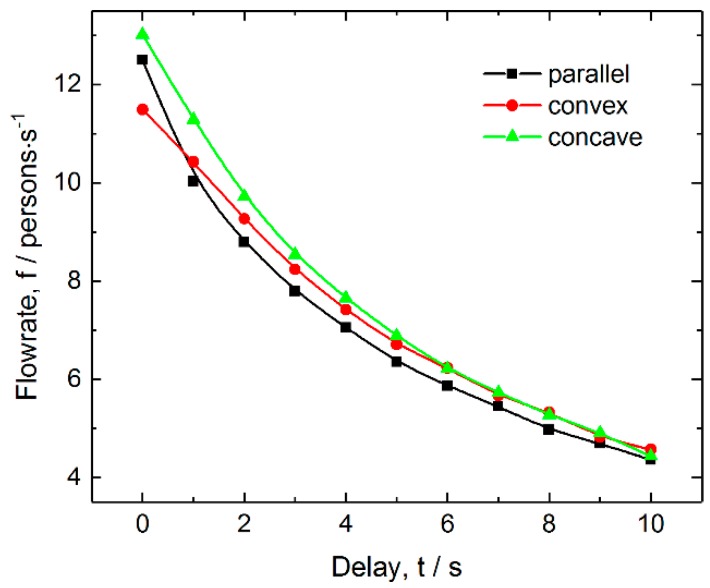
Total flowrate as a function of delay for the three layouts of inspection points. The density of pedestrian and failure probability are 3.5 persons/m^2^ and 0.08, respectively.

Figure 5 shows the total flowrate as a function of delay for the three layouts. The figure reveals that all the flowrates decay nonlinearly as the delay increases. This is because the utilization of the exits decreases when the delay increases. In addition, the flowrate of pedestrians for the parallel layout decays most rapidly among the three layouts, which also indicates that the parallel layout has the weakest resistance to delay because of ticket-checking failure. The concave layout always has the largest flowrate among the three layouts in the considered range of delays. It is noted that the relations of flowrate with the delay for the three layouts are very similar to those with failure probability. The corresponding density distributions of pedestrian also have obvious similarity.

### 3.2. Passing Safety

In reality, when two or several pedestrians compete for the same position, there would be a collision between the pedestrians, increasing the risk of injury or crowd disturbances. In the previous work of Lin et al. [38], it was found that the competitive behavior of pedestrians at the exit may cause force localization and compressive asphyxia of people. In the CA simulation, such behavior is associated with two or three pedestrians completing for one cell when updating their positions. In our previous work, we introduced competitive frequency to evaluate the risk level of such behavior [24]. In this work, we use competitive pedestrian-time and competitive frequency to evaluate the risk level, which are defined as the pedestrian-time and times of competitive behavior in unit time and area, respectively. The competitive frequency includes two-pedestrian and three-pedestrian competitive frequencies. A lower competitive pedestrian-time and a competitive frequency represent a higher risk.

#### 3.2.1. Effects of Pedestrian Density on Competitive Level

**Figure 6:**
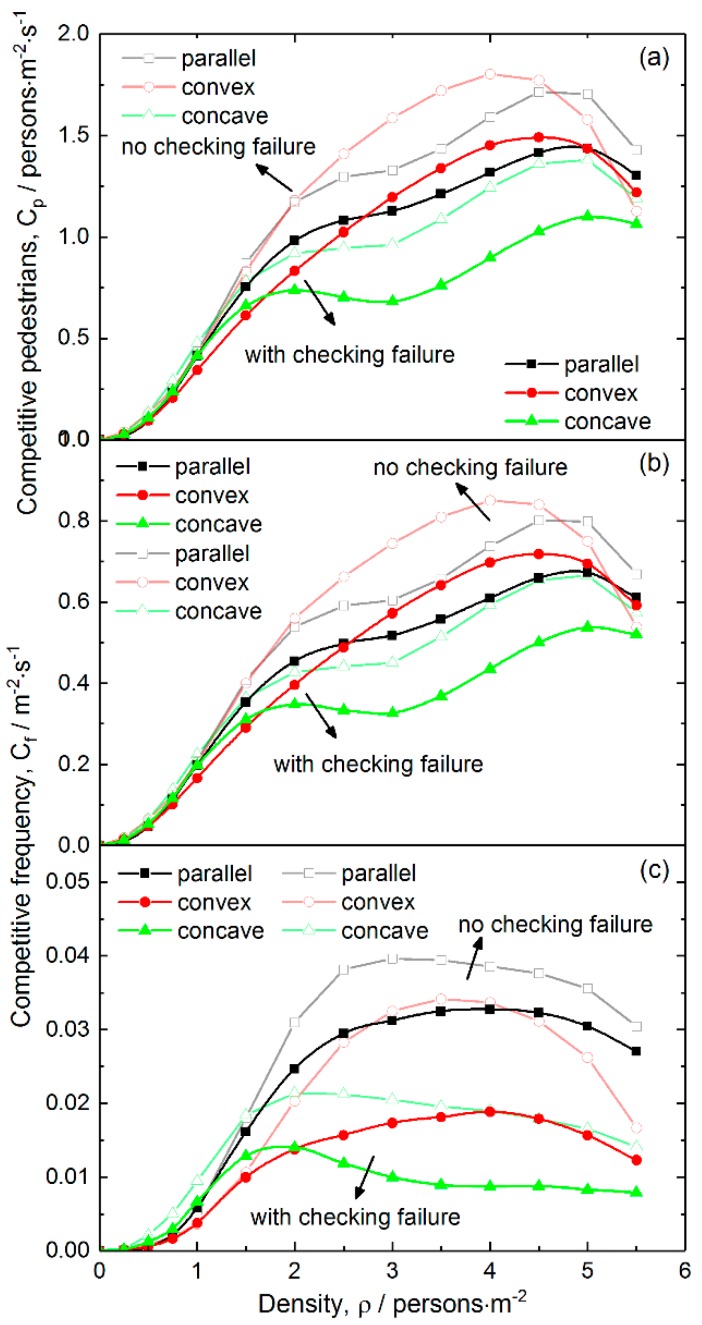
Competitive intensity as a function of pedestrian density for the three layouts of inspection points: (**a**) Competitive pedestrian-time, (**b**) two-person competitive frequency and (**c**) three-person competitive frequency. The checking failure probability and delay are 0.08 and 3 s, respectively. The open symbols are the results obtained under conditions without checking failure.

Figure 6 shows the competitive pedestrian-time and competitive frequency under conditions of various pedestrian density and fixed failure probability for the three layouts. For comparison, the results for the cases without checking failure are also shown in the figure. 

Figure 6a reveals that the concave layout has the smallest competitive pedestrian-time in the medium and high-density range among the three layouts. This result indicates that the concave layout has the best safety. In the low-density range, there is no obvious difference in competitive pedestrian-time between the cases with and without checking failure. However, when the density is above 1.5 persons/m^2^, the difference becomes increasingly apparent. Such a difference is largest in the medium density range, and it slightly goes down in the high-density range. These results suggest that the occurrence of checking failure is able to alleviate the competition between pedestrians to some extent, and decreases the risk of crowd disturbance. Such an effect comes from the fact that the checking failure temporarily blocks the exit and induces pedestrian congestion near the front of the exit, which decreases the difference of near-exit local density between the exits and the frequency of changing target exits (see Figure 4). As to the slight decrease of competitive difference in the high-density range, it is because most cells are occupied and few cells are available for competition. Instead, pedestrians have to wait. 

**Figure 7:**
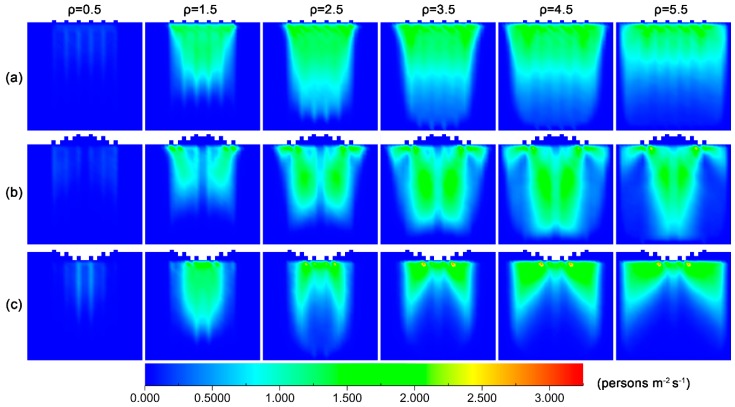
Distribution of competitive pedestrian-time at different pedestrian densities for the three layouts of inspection points: (**a**) parallel, (**b**) convex and (**c**) concave. The checking failure probability and delay are 0.08 and 3 s, respectively. The unit of pedestrian density is persons/m^2^.

To further understand the effects of pedestrian density on the competitive behavior, Figure 6b,c shows the two-pedestrian and three-pedestrian competitive frequencies for the three layouts as a function of pedestrian density, respectively. It can be found from the figures that the three-pedestrian competitive frequency is far smaller than the corresponding two-pedestrian one. Notably, the two-pedestrian competitive frequency for the concave layout first shows a dropping tendency when the density is above 2.0 persons/m^2^, and then goes up. However, the three-pedestrian one always drops when the density is above 2.0 persons/m^2^ until it reaches an equilibrium level. For the parallel and convex layouts, both the two-pedestrian and three-pedestrian frequencies increase with the density in the medium density range. To get an insight into the effects of pedestrian density on competitive behavior, we calculated the distribution of competitive pedestrian-time in the scene for the three layouts at various pedestrian densities, and is shown in Figure 7. 

The figure indicates that with a specific failure probability, the distribution of competitive pedestrian-time in the scene varies greatly between the layouts. In the medium and high density range, the competitive pedestrian-time for the parallel layout is mainly distributed in the front region of the exits, that for the convex layout is mainly distributed in the two sides near the middle part of the scene, and that for the concave layout is mainly distributed in the two sides of the front part. Of note that there is intensive competition near the two side exits for the convex layout, and it is small near the middle exits. For the concave layout, in contrast, there is intensive competition near the two middle exits and little competition near the side exits. In addition, with the increase in pedestrian density, the distribution of competitive pedestrian-time for the parallel layout first diffuses from the near-exit region to the rear part of the scene, and then is compressed to the front part of the scene. For the convex layout, the distribution of competitive pedestrian-time always diffuses from the front region to the rear part of the scene with the increase in pedestrian density, until the density is above 4.5 persons/m^2^ it is slightly compressed from the two sides to the middle part. It is interesting that for the concave layout, the distribution of competitive pedestrian-time is mainly distributed in the middle region of the front part in the low-density region. However, with the increase in pedestrian density, it is divided into two parts and diffuses from the middle region to the two sides, which shows a transition to a new distribution mode. This result agrees with the finding that the competitive pedestrian-time shows a slight decrease when the pedestrian density is above 2.0 m^2^. This phenomenon may be because the pedestrians tend to select the nearest middle exits as their targets in the low-density range. However with the increase in pedestrian density, pedestrian congestion appears at the middle exits, and the pedestrians turn to select the side exits as their targets.

#### 3.2.2. Effects of Failure Probability on the Competition Level

**Figure 8:**
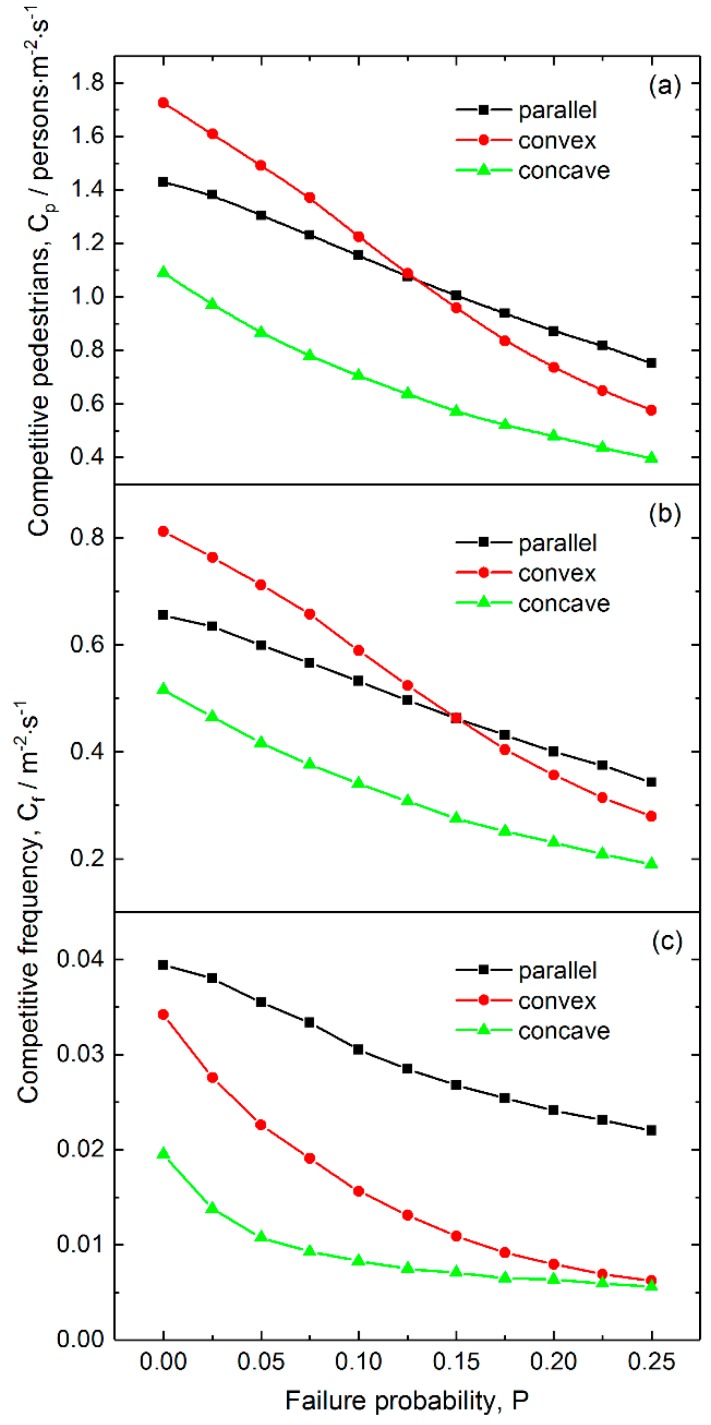
Competitive intensity as a function of checking failure probability for the three layouts of inspection points: (**a**) Competitive pedestrian-time, (**b**) two-person competitive frequency and (**c**) three-person competitive frequency. The pedestrian density and average delay are 3.5 persons/m^2^ and 3 s, respectively.

Figure 8 shows the pedestrian-time competition and frequency for various failure probabilities. Figure 8a reveals that all the competitive pedestrian-times decrease nearly linearly with increasing failure probability, among which that for the concave layout is the smallest in the whole considered range of failure probabilities, and that for the convex layout decreases most quickly. Figure 8b indicates that the two-pedestrian competitive frequency has a similar dependence on failure probability as competitive pedestrian-time. The concave layout has the smallest competitive frequency. 

Figure 8c reveals that the three-pedestrian competitive frequency decreases nonlinearly with increasing failure probability for the three layouts, among which that for the concave layout is the smallest, and that for the parallel layout is the largest. These results suggest that a proper probability of ticket-checking failure would give rise to less competitive behavior between pedestrians. Therefore, maintaining a certain level of failure probability is an effective approach to reduce competitive events and to improve the ordering of pedestrians passing through the exits.

**Figure 9:**
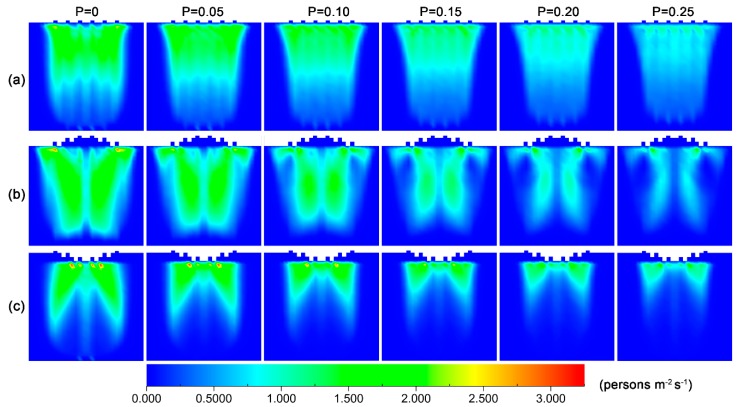
Distribution of competitive pedestrian-time at different checking failure probabilities for the three layouts of inspection points: (**a**) parallel, (**b**) convex and (**c**) concave. The checking failure probability and delay are 0.08 and 3 s, respectively. The unit of pedestrian density is persons/m^2^.

To gain an insight into the effects of failure probability on competitive behavior, we calculated the distribution of competitive pedestrian-time in the scene for the three layouts at various pedestrian densities, and the results are shown in Figure 9. The figure reveals that there is no obvious change in the distribution regions of the competitive pedestrian-times for the three layouts when increasing failure probability, but their magnitudes gradually decrease. In combination with the density distribution of pedestrian in Figure 5, this change may result from the congestion of pedestrians in front of the exits because of checking failure. The congestion not only makes the pedestrians wait, but also reduces the number of empty cells near the exits. Both these aspects contribute to the relief of competition between pedestrians. 

#### 3.2.3. Effects of Delay on Competitive Level

**Figure 10:**
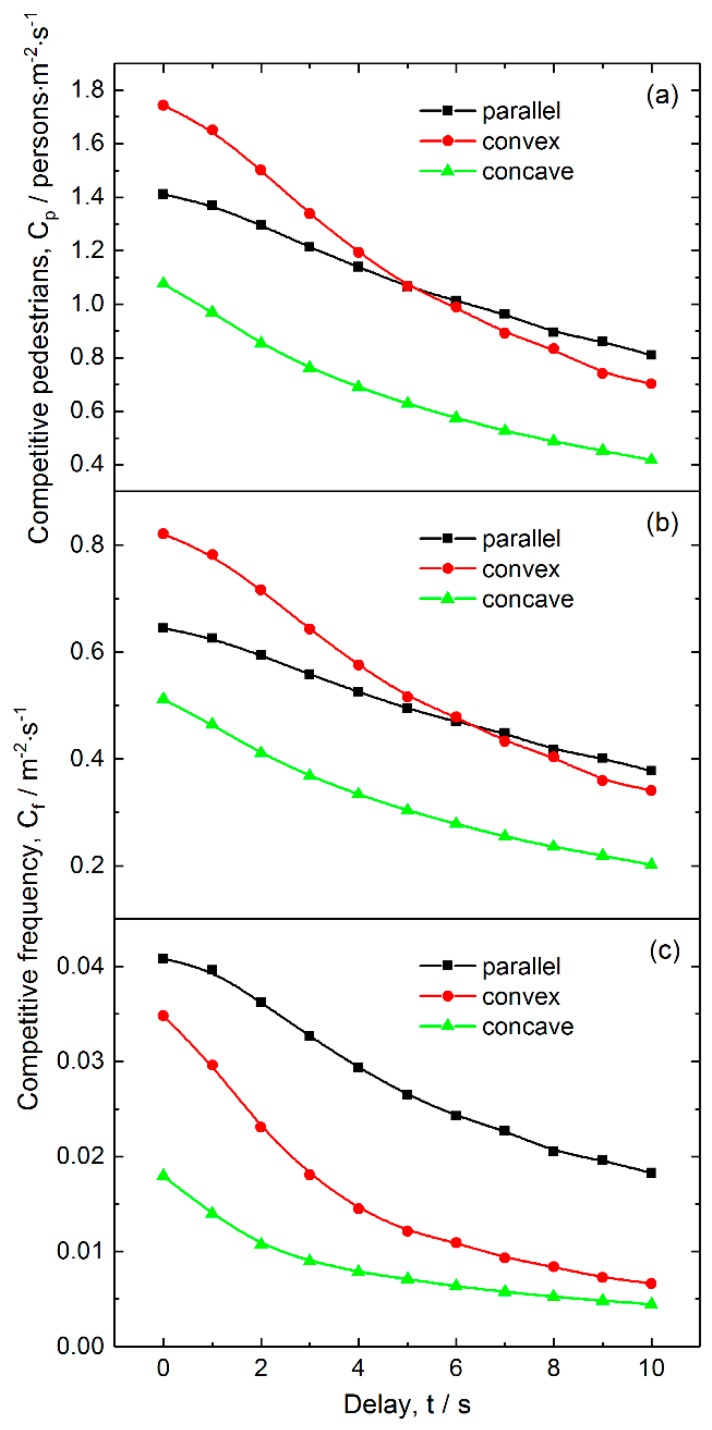
Competitive intensity as a function of average delay for the three layouts of inspection points: (**a**) Competitive pedestrian-time, (**b**) two-person competitive frequency and (**c**) three-person competitive frequency. The pedestrian density and average delay are 3.5 persons/m^2^ and 3 s, respectively.

The competitive pedestrian-time and frequency at various delays are shown in Figure 10. Figure 10a indicates that the competitive pedestrian-time decreases when increasing delay, and that it is much less for the concave layout than those for the left two layouts in the whole considered range of delay. Figure 10b reveals that the competitive pedestrian-time and two-pedestrian competitive frequency have similar dependences on delay, and the concave layout has the lowest two-pedestrian competitive frequency. Figure 10c shows that the three-pedestrian competitive frequency nonlinearly decreases when the delay increases for the three layouts, among which that for the concave layout is the lowest, and that for the parallel layout is the highest. These results indicate that a proper delay because of ticket-checking failure is able to relieve the competitive behavior between pedestrians to some extent. Therefore, keeping a certain period of delay is also an effective approach to reduce competitive events and to improve the ordering of pedestrians when passing through the exits. However, it should be noted that in terms of psychology, excessive delay may wear down the patience of pedestrians, causing fidgety moods and crowd disturbances.

### 3.3. Equivalence of Failure Probability and Delay

It can be easily found from the above results that failure probability and delay have similar effects on passing efficiency and competitive behavior. We defined the product of delay and failure probability as the integrated average delay. To further check whether these two factors are equivalent in affecting passing efficiency and competitive behavior, we simulated three independent cases for each integrated average delay as listed in Table 1. We take total flowrate and competitive pedestrian-time as the references of their equivalence. The equivalence of these three cases can be evaluated by the coefficient of variation, which is expressed as:

(9) $$C_{v}=S/\mu \times 100\%$$

where S and μ are the standard deviation and mean of total flowrate or competitive pedestrian-time for the three samples, respectively. A small coefficient of variation represents a high equivalence.

**Figure 11:**
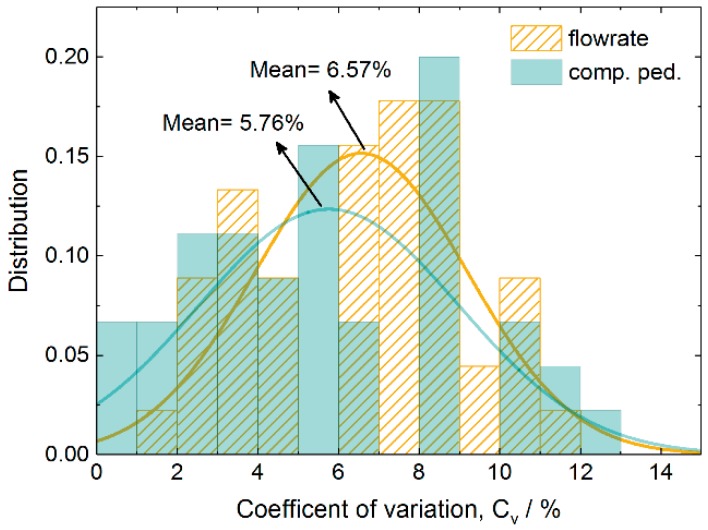
Distributions of variation coefficient for the considered cases with various checking failure probabilities and delay times in terms of total flowrate and competitive pedestrian-time. The lines are the corresponding Gaussian distributions.

Figure 11 shows the distributions of the variation coefficient of total flowrate and competitive pedestrian-time for the cases listed in Table 1. The distributions can be fitted well by a Gaussian distribution with means as small as 6.57% and 5.76% for the total flowrate and competitive pedestrian-time, respectively. These results demonstrate that the influences of failure probability and delay on the process of pedestrians passing through the multi-exit inspection points are highly equivalent to each other in terms of total flowrate and competitive pedestrian-time. 

**Figure 12:**
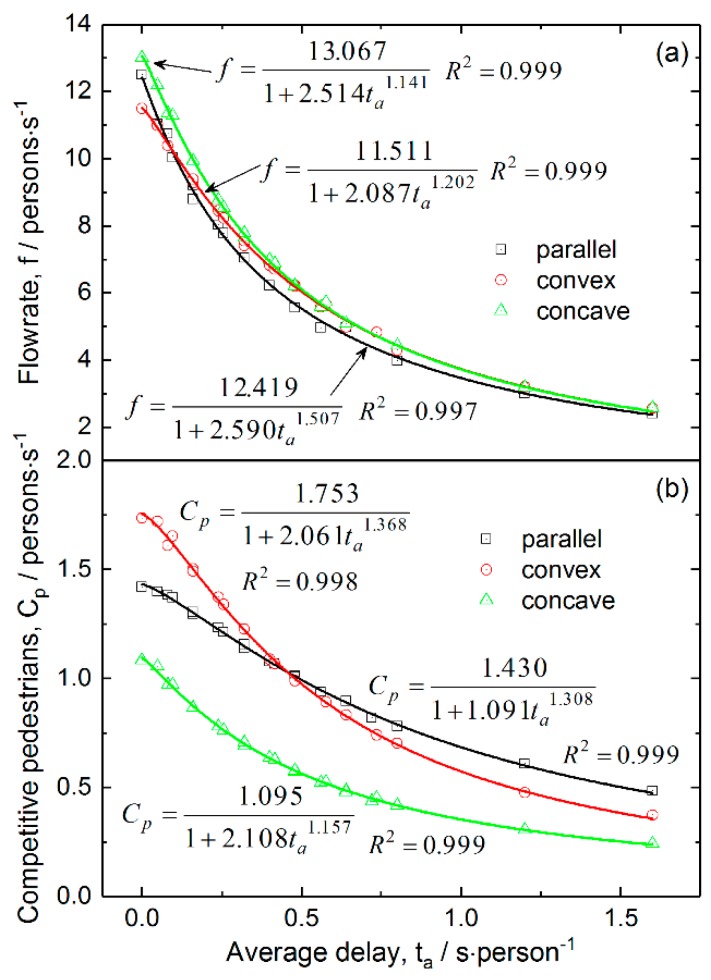
Dependence of (**a**) total pedestrian flowrate and (**b**) competitive pedestrian-time on integrated average delay for the three layouts. The lines are fitted from the data with the proposed model.

To quantitatively describe the dependence of the total flowrate and competitive pedestrian-time on the integrated average delay time, we defined the model:

(10) $$f\;(or\;C_{p})=\frac{a}{1+b\cdot t_{a}{}^{c}}$$

where a, b and c are constants, and f, $C_{p}$ and $t_{a}$ represent the total flowrate, competitive pedestrian-time and integrated average delay, respectively. According to this model, a is the flowrate or competitive pedestrian-time under the condition without checking failure, and when the integrated average delay approaches infinite, the function will be zero. This model is derived from the modification of the ideal case after considering checking failure in real situations. The fits of the total flowrate and competitive pedestrian-time against the integrated average delay for the three layouts are shown in Figure 12. The results reveal that the coefficients of determination ($R^{2}$) are extremely high, which suggests that both the dependences of total flowrate and competitive pedestrian-time on the integrated average delay can be well described by the proposed model. The fits of data in the figure also indicate that the concave layout has the largest total flowrate and lowest competitive level among the three layouts in the considered range of integrated average delay and even in ideal situations. 

## 4. Conclusions

In this work, we investigated the effects of ticket-checking failure on the dynamics of pedestrians at the multi-exit inspection points with various layouts by microscopic simulations with the modified CA model. Parallel, convex and concave layouts were considered and analyses were carried out in terms of passing efficiency and safety. The obtained results indicate that in the medium and high pedestrian density range, the occurrence of ticket-checking failure would obviously reduce passing efficiency, and its reducing degree increases nonlinearly with failure probability and delay. However, the behavior of ticket-checking failure would also alleviate the competition between pedestrians for walking paths, which can promote the passing safety of pedestrians. Such an effect is particularly obvious in the medium and high-density pedestrian range. The level of competitive behavior between pedestrians decreases when the failure probability and delay increase. It is also found that the failure probability and corresponding delay are equivalent in affecting passing efficiency and competitive behavior, and can be integrated as an average delay. A fitted equation was proposed for the dependence of passing efficiency and safety on the average delay. In reality with the existence of ticket-checking failure, the concave layout is the best choice compared with the parallel and convex ones, as there is a much lower competitive level between pedestrians.

## Figures and Tables

**Table 1:** Cases with various checking failure probabilities and delay times for the verification of equivalence.

Group No.	Case No.	1		2		3	
	Prob. × Delay/s	Prob.	Delay/s	Prob.	Delay/s	Prob.	Delay/s
1	0.048	0.010	4.800	0.030	1.600	0.120	0.400
2	0.480	0.060	8.000	0.100	4.800	0.200	2.400
3	0.800	0.100	8.000	0.125	6.400	0.250	3.200
4	1.200	0.100	12.000	0.150	8.000	0.250	4.800
5	1.600	0.100	16.000	0.160	10.000	0.250	6.400
